# Biomechanical Changes in Kyphotic Cervical Spine After Anterior Cervical Discectomy and Fusion with Different Degrees of Correction

**DOI:** 10.3390/bioengineering12030213

**Published:** 2025-02-20

**Authors:** Hongyu Chen, Xu Ma, Shengfa Pan, Li Zhang, Yanbin Zhao, Xin Chen, Yu Sun, Feifei Zhou

**Affiliations:** 1 Department of Orthopaedics, Peking University Third Hospital, Beijing 100191, China; hychen0727@163.com (H.C.); panshengfa@vip.sina.com (S.P.); drzhli@163.com (L.Z.); dageb@aliyun.com (Y.Z.); chenxinbjmu@hotmail.com (X.C.); sunyuor@vip.sina.com (Y.S.); 2 Engineering Research Center of Bone and Joint Precision Medicine, Beijing 100191, China; 3 Beijing Key Laboratory of Spinal Disease Research, Beijing 100191, China; 4 Key Laboratory for Biomechanics and Mechanobiology of Ministry of Education, Beijing 100191, China; maxu1@foxmail.com; 5 Advanced Innovation Center for Biomedical Engineering, School of Biological Science and Medical Engineering, Beihang University, Beijing 100191, China

**Keywords:** cervical kyphosis, correction, biomechanical, finite element

## Abstract

Cervical kyphosis is a debilitating disease, and its surgical treatment involves correction to restore sagittal alignment. Few studies have explored the appropriate degree of correction, and the biomechanical impact of correction on the cervical spine is still unclear. This study aimed to compare the biomechanical changes in the cervical spine after different degrees of correction by two-level anterior cervical discectomy and fusion (ACDF). Three-dimensional finite element (FE) models of the intact cervical spine (C2–C7) with normal physiological lordosis and kyphosis were constructed. Based on the kyphotic model, three two-level ACDF in C4–6 surgical models were developed: (1) non-correction: only the intervertebral heights were restored; (2) partial correction: the cervical curvature was adjusted to straighten; (3) complete correction: the cervical curvature was adjusted to physiological lordosis. A pure moment of 1.0 Nm combined with a follower load of 73.6 N was applied to the C2 vertebra to simulate flexion, extension, lateral bending, and axial rotation. The stress of vertical bodies and facet joints, intradiscal pressure (IDP), and the overall ROMs of all models were computed. The peak von Mises stress on the upper (C4) and lower (C6) instrumented vertebral bodies in the kyphotic model was greater than that of the physiological lordosis model, with the exception of C6 under lateral bending. The maximum stress was observed in C4 during lateral bending after complete correction, which increased by 145% compared to preoperative von Mises stress. For the middle (C5) instrumented vertebral body, the peak von Mises stress increased after surgery. The maximum stress was observed in partial correction during flexion. Compared to physiological lordosis, the peak von Mises stress on the facet joints in kyphotic segments was lower; however, it was higher in the adjacent segments, except C4/5 in extension. The stress on the facet joints in kyphotic segments decreased, with the most significant decrease observed in partial correction. The IDPs in adjacent segments, except for C6/7 in flexion, showed no significant difference before and after surgery. Additionally, correction seemed to have little impact on IDPs in adjacent segments. In conclusion, for the treatment of cervical kyphosis with two-level ACDF, complete correction resulted in the highest peak von Mises stress on the upper instrumented vertebral body. Partial correction mitigated von Mises stress within the facet joints in kyphotic segments, albeit at the expense of high von Mises stress on the middle instrumented vertebral body.

## 1. Introduction

The cervical spine, supporting the mass of the head, compensates for thoracic kyphosis by exaggerating the sagittal lordotic curvature to maintain horizontal gaze [1,2]. With the physiological aging process, the cervical spine sometimes has kyphotic changes due to disc and facet joint degeneration [3,4]. Previous studies have documented the relationship between cervical sagittal malalignment and disability and functional impairment by health-related quality-of-life (HRQOL) measures [5,6]. Mild cervical kyphosis in individuals with minimal neurologic compromise can often be managed conservatively. However, significant deformity and neurologic deficits can only be corrected with surgical intervention [7].

Anterior cervical discectomy and fusion (ACDF) is a relatively facile and effective strategy for the correction of cervical kyphosis, which mainly decompresses and corrects through the intervertebral space [8,9]. Increasing or maintaining cervical lordosis is a commonly accepted goal in the surgical treatment of cervical spondylosis [10]. However, the association between sagittal radiographic parameters and clinical improvement is not clear, and there is still disagreement about the optimal degree of sagittal alignment [11,12,13,14]. For non-severe cervical kyphosis, whether to correct and the degree of correction often depends on the surgeon’s experience and preference. Previous clinical observations seem to suggest that anterior surgical correction of cervical kyphosis does not require complete correction [12,15]. However, some scholars worry that if the cervical sagittal alignment is not restored, the degree of deformity may be aggravated, or adjacent segment degeneration (ASD) may occur [16,17].

Finite element (FE) analysis is a crucial in vitro experiment that can evaluate the biomechanical performance of the cervical spine before and after surgery through realistic simulation. Some previous studies have investigated the biomechanical effects of ACDF [18,19]. However, the existing literature on the biomechanical outcomes of ACDF in the context of cervical kyphosis is limited.

In recent years, several studies have focused on the development and validation of FE models for the cervical spine, aiming to understand the effects of different surgical interventions on spinal stability and load distribution [18,19,20,21]. For instance, studies by Guo et al. [18] and Liang et al. [21] predicted the biomechanical response of the cervical spine in various surgical treatments through FE models, including ACDF. These models have been instrumental in identifying the factors that contribute to the success or failure of spinal fusion procedures.

Despite these advancements, there have been no studies analyzing the biomechanical changes in the cervical spine after different degrees of correction of cervical kyphosis achieved by ACDF. Therefore, the present study established cervical FE models of normal, kyphotic, and different degrees of kyphotic correction by ACDF and evaluated the biomechanical differences between them. To the best of the authors’ knowledge, this is the first biomechanical study to explore ACDF for the treatment of cervical kyphosis. The objective of the study was to find an optimal degree of ACDF correction for cervical kyphosis from a biomechanical perspective.

## 2. Materials and Methods

A three-dimensional FE model of C2–C7 was reconstructed based on computed tomography (CT) scans, with 1 mm intervals (Dual Source CT; Siemens, Munich, Germany), of a female patient (age: 35; height: 166 cm; weight: 69 kg; BMI: 25.0) with degenerative non-rigid cervical kyphosis; it was defined as the Kyphosis FE model. Then, a healthy female volunteer of similar age and BMI (age: 38; height: 169 cm; body mass: 73 kg; BMI: 25.6) was selected, and another FE model was reconstructed using her CT scan; this model was defined as the Normal FE model. The study was approved by the Peking University Third Hospital Medical Science Research Ethics Committee (Ethical batch number: M2022752).

### 2.1. Generation of Cervical Spine Model and Instrument

The computed tomography data were imported into Mimics software v21.0 (Materialise, Inc., Leuven, Belgium) in DICOM format to reconstruct the geometry of the cervical spine model. The initial models were surface-smoothed using Geomagic Studio 2012 (Geomagic Inc., Charlotte, NC, USA). The intervertebral disc geometries were constructed by filling the intervertebral space and connecting the adjacent vertebral bodies. Afterwards, these components of FE models were meshed in Hypermesh v16.0 (Altair Engineering, Troy, MI, USA). Finally, the boundary conditions of the prepared model were set using ABAQUS 2021 (Dassault Systems Corporation, Waltham, MA, USA; version 2021).

The cancellous bone regions of the vertebrae were set as solid elements. A 0.4 mm thick shell consisting of cortical bone and endplates covered the cancellous bone [22]. The intervertebral disc was composed of nucleus pulposus and annulus fibrosus with a volume ratio of 6:4 [22]. The upper and lower surfaces of the nucleus pulposus and annulus fibrosus were anchored to the endplate, forcing all translational and rotational degrees of freedom (DOF) to be the same. The cervical ligaments, including the anterior longitudinal ligament (ALL), posterior longitudinal ligament (PLL), ligamentum flavum (LF), interspinous ligament (ISL), and supraspinal ligament (SSL), were developed using tension-only rod elements and attached to the corresponding vertebrae.

**Figure 1:**
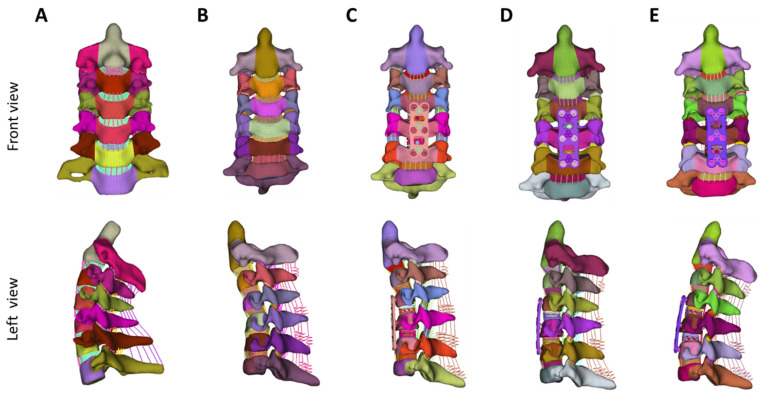
Three-dimensional FE models of cervical spine (C2–C7). (**A**) Physiological lordosis; (**B**) kyphosis; (**C**) non-correction; (**D**) partial correction; (**E**) complete correction.

The local cervical kyphosis in the patient was mainly located in the C4–6 segment; therefore, the surgery intervened above the two segments. The discs and ALL of C4–5 and C5–6 were completely resected on the Kyphosis FE model, and two cages were placed at each cavity to simulate two-level anterior cervical discectomy and fusion (ACDF). The interbody fusion device was composed of a titanium plate, two cages capable of adjusting the height or the angle of the upper and lower planes (Naton, Beijing, China), and six self-tapping screws. Three different surgical correction methods were set up: (1) non-correction: restoring the intervertebral height without adjusting the cervical curvature; (2) neutral: restoring the intervertebral height and adjusting the cervical curvature to straighten (C2–7 Cobb angle = 0°); (3) lordosis: restoring the intervertebral height and adjusting the cervical curvature to physiological lordosis (C2–7 Cobb angle = 16° [23,24]) (Figure 1). Each correction method was achieved by adjusting the height and angle of the upper and lower planes of the two cages. By adjusting the cages to the appropriate parameters, these upper and lower surfaces were made to fit the adjacent endplates.

The contact surfaces between the cages and endplates were defined as Tie contact, and common nodes were set between the screws and the vertebral bodies to simulate rigid fusion and full osseointegration [25,26]. All material properties and element types of the components of the cervical spine and implants are shown in Table 1.

### 2.2. Boundary and Loading Conditions

**Figure 2:**
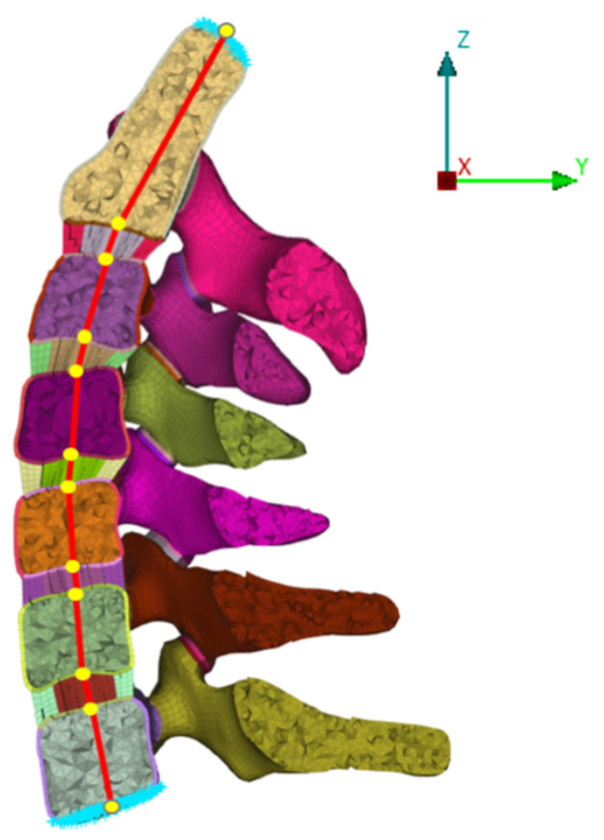
Schematic diagram of loading condition in FE models.

Coupling points were arranged on the upper surface, and the lower surface of all segmental vertebral bodies, and the coupling points were sequentially connected through a connecting unit to simulate a physiological axis. A compressive load of 73.6 N was applied to the attachment unit of each segment to simulate the weight of the head and muscle force. A 1.0 N m moment was performed around the X, Y, and Z axes at the top of C2 to simulate flexion, extension, lateral bending, or axial rotation (Figure 2). The support was coupled to the center of the C7 lower endplate and constrained in all directions. The universal contact was set, and the coefficient of friction was set to 0.01. The range of motion (ROM) was calculated based on the relative motions of the markers of each vertebra in each motion model. The ROM of the Normal FE model under all moments was compared with previously published data [25,26,27,28] to validate the model. In the above five FE models, the stress condition of vertebral bodies and facet joints, intradiscal pressure (IDP), and the overall ROMs were tested under all motion conditions.

## 3. Results

### 3.1. Validation of the Cervical FE Model

**Figure 3:**
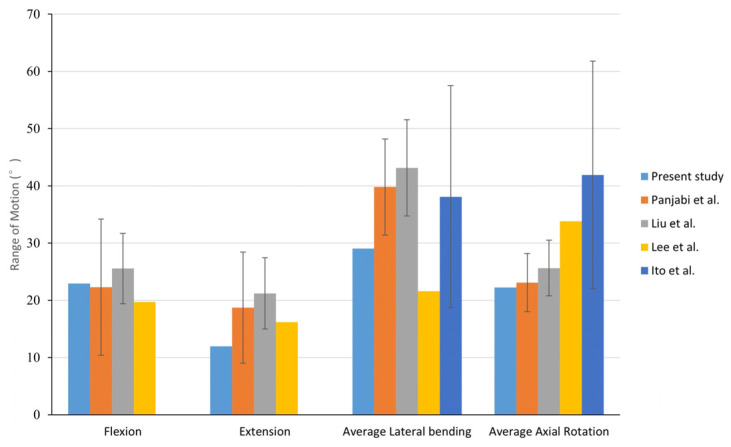
Comparison of C2–C7 ROM with previously published studies [27,28,29,30].

The Normal FE model was compared with four previous biomechanical studies [27,28,29,30] to assess its validity. The predicted cervical ROM of the flexion–extension, lateral bending, and axial rotation of the cervical spine model were in good agreement with those of previous experiments (Figure 3).

### 3.2. Peak von Mises Stress of Instrumented Vertebral Bodies

**Figure 4:**
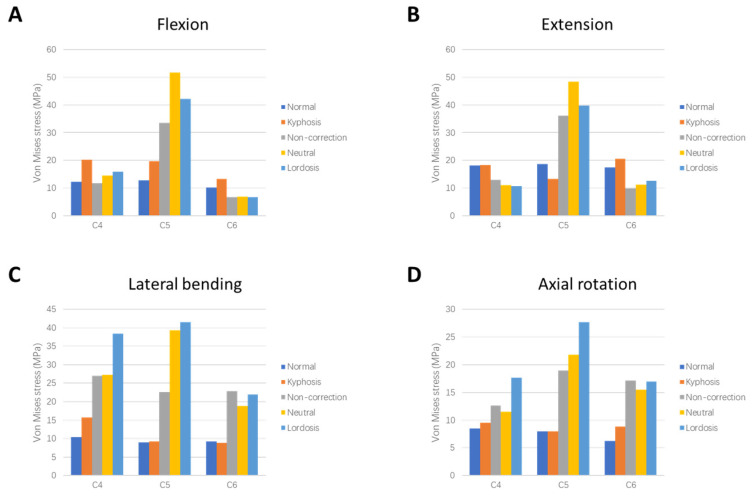
Comparison of peak von Mises stress of instrumented vertebral bodies between different models in (**A**) flexion, (**B**) extension, (**C**) lateral bending, and (**D**) axial rotation.

The peak von Mises stress (hereafter referred to as stress) models of instrumented vertebral bodies in cervical motion are shown in Figure 4. For the upper and lower vertebral bodies (C4 and C6), the stress of the Kyphosis model was greater than that of the Normal model under the working conditions of forward flexion, backward extension, lateral bending, and axial rotation, except for the C6 vertebral body under lateral bending. The three surgical models (the Non-correction model, the Neutral model, and the Lordosis model) exhibited higher stresses on the superior and inferior vertebral bodies during lateral bending and axial rotation compared to the Kyphosis model. The maximum stress of the upper and lower vertebral bodies occurred during lateral bending of C4 in the Lordosis model, which was 145% higher than that of the Kyphosis model. Peak stress on the superior and inferior vertebral bodies during flexion was reduced in the three surgical models compared to the Kyphosis model.

For the middle vertebral body (C5), the stress in surgical models was significantly higher than that in the Kyphosis model. The maximum stress (51.6 MPa) was observed in the Neutral model during flexion. Among the three surgical models, the C5 peak von Mises stress in the Non-correction model was the smallest.

### 3.3. Von Mises Stress of Facet Joints

**Figure 5:**
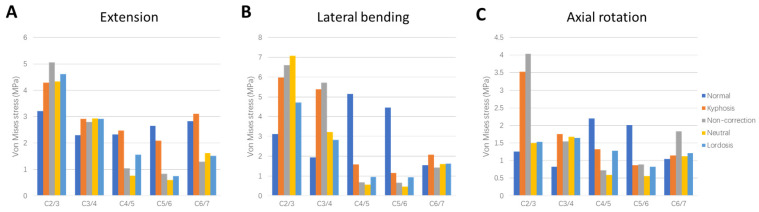
Comparison of peak von Mises stress of facet joints between different models in (**A**) extension, (**B**) lateral bending, and (**C**) axial rotation.

The peak von Mises stress of facet joints in various segments of the cervical spine under extension, lateral bending, and axial rotation are shown in Figure 5. Compared to the Normal model, the stress of facet joints in the adjacent segments (C2/3, C3/4, and C6/7) of the Kyphosis model increased. At the extension position, the stress of facet joints increased by 35.3%, 26.7%, and 9.8% at C2/3, C3/4, and C6/7, respectively. On the contrary, the stress of the facet joint in the Kyphosis model in the kyphosis segments (C4/5 and C5/6) model was smaller compared to the Normal model, except C4/5 in extension.

Compared with the Kyphosis model, the stress of facet joints in the kyphosis segments of the three surgical models decreased. Among them, the Neutral model had the most obvious decrease: C4/5 decreased by 69.6%, 65.0%, and 55.0% in extension, lateral bending, and axial rotation, respectively; C5/6 decreased by 71.2%, 60.2%, and 35.5% in extension, lateral bending, and axial rotation, respectively. In the three surgical models, no method with generally lower stress in the facet joints of adjacent segments was observed.

### 3.4. Intradiscal Pressure in Adjacent Segments

**Figure 6:**
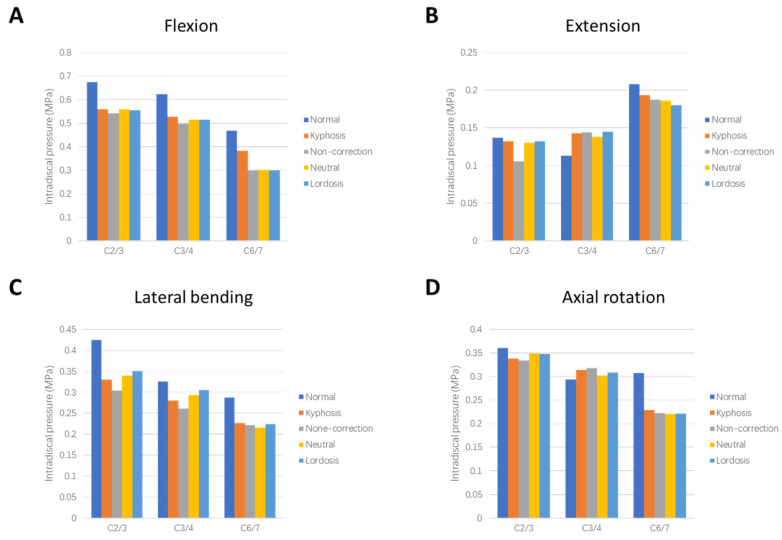
Comparison of IDPs at adjacent levels between different models in (**A**) flexion, (**B**) extension, (**C**) lateral bending, and (**D**) axial rotation.

**Figure 7:**
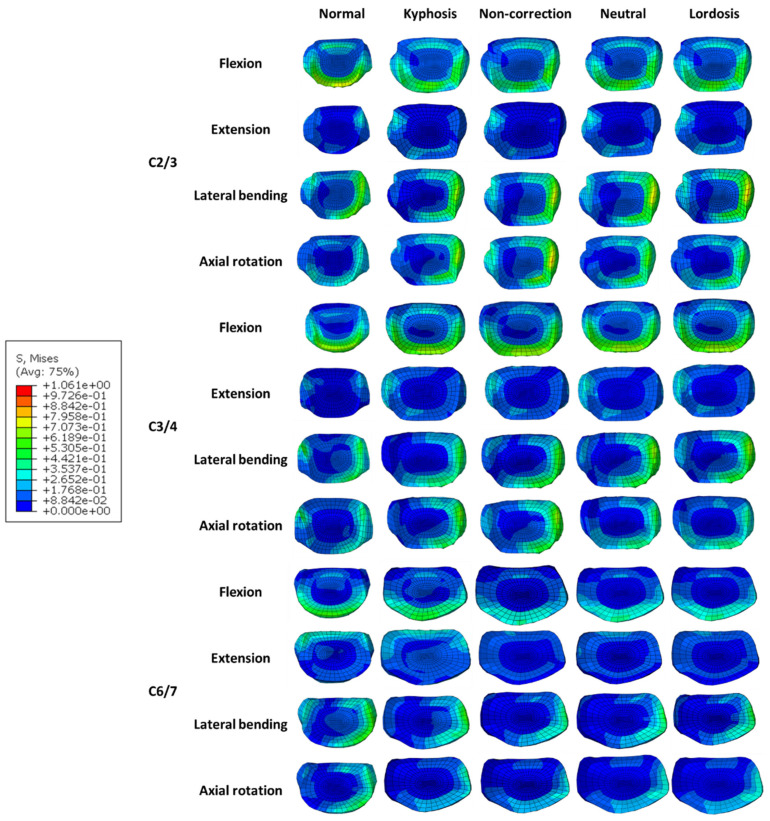
The stress distribution of intervertebral discs in adjacent segments for different models under four motion conditions.

Intradiscal pressure (IDP) measurements of the supra-adjacent (C2/3 and C3/4) and infra-adjacent (C6/7) segments are shown in Figure 6. The maximum IDPs were noted at the end of the flexion moment in C2/3. The IDPs of the Normal model were smaller than those of the Kyphosis model in C3/4 of the extension and axial rotation moment, while the results are opposite in the other conditions. After surgery, the IDPs changed little (<10%) except C6/7 in flexion. There was no significant difference in adjacent segments IDPs among the three surgical models under different working conditions. The stress distributions of the intervertebral discs in adjacent segments are shown in Figure 7. The intervertebral disc stress distributions of the five models were similar. In the C6/7 segment, the overall stress values of the three surgical models were lower than that of the Kyphosis model.

### 3.5. ROMs of C2–7

**Figure 8:**
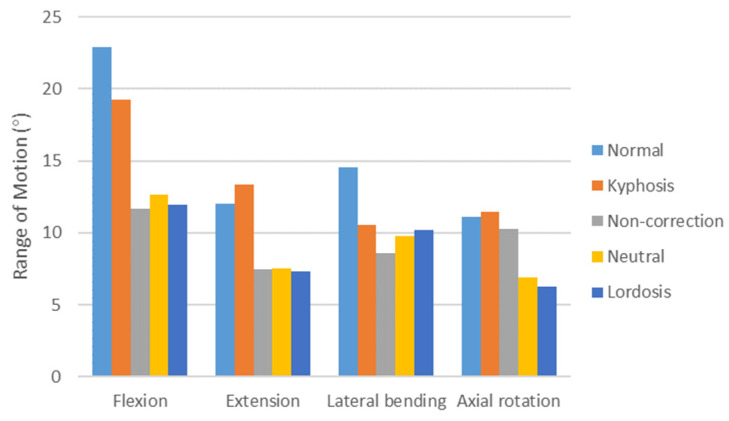
Comparison of ROMs between different models under four motion conditions.

The ROMs of C2–7 in the Normal model, the Kyphosis model, and the surgical models are shown in Figure 8. In flexion and lateral bending, the ROM of the Kyphosis model was reduced by 16.2% and 27.1%, respectively, compared with the Normal model. In extension and axial rotation, the ROM of the Kyphosis model was slightly greater than that of the Normal model.

The ROMs of the three surgical models were less than that of the Kyphosis model in all working conditions. The ROMs of the Non-correction model, Neutral model, and Lordosis model were, respectively, 11.7°, 12.7°, and 12.0° in flexion; 7.4°, 7.5°, and 7.3° in extension; 8.6°, 9.8°, and 10.2° in lateral bending; and 10.2°, 6.9°, and 6.2° in axial rotation.

## 4. Discussion

The surgical treatment of cervical kyphosis has always been the focus of discussion in spinal surgery, which involves decompression and correction, and the choice of the degree of correction is still controversial [12,14]. Several studies have reported on the relationship between sagittal alignment recovery and clinical outcomes in cervical kyphosis. Hu et al. [16] investigated patients receiving one and two-level ACDF and found that patients who restored cervical alignment had significantly greater improvement in NDI and relatively better improvement in neck pain. Patients with unchanged cervical kyphosis tended to have a higher incidence of ASD. Tang et al. [31] evaluated patients undergoing multilevel posterior cervical fusion and found a significant correlation between C2–C7 SVA and NDI scores. Cervical sagittal malalignment is closely related to pathomechanics [32]. However, to the best of our knowledge, few studies have reported the biomechanical effects of surgical treatment of cervical kyphosis. The present study simulated and compared cervical spine biomechanics of physiological lordosis, kyphosis, and different degrees of correction through two levels. We found that the kyphosis cervical spine had lower adjacent segment facet joint stress and greater ROM values than the physiological lordosis spine. However, at the peak von Mises stress of vertebral bodies and facet joints in kyphotic segments, and IDPs in adjacent segments, the physiological lordosis did not confer a biomechanical advantage over kyphosis. Among the three surgery methods of non-correction, partial correction, and complete correction, the partial correction had the lowest peak von Mises stress in the facet joints of the instrumented segments, but the cost was higher stress in the middle instrumented vertebral body.

The load on the vertebral body is transmitted downward through the diffusion of a rigid structure composed of cortical bone [33]. The results of this study suggest that the kyphotic cervical spine made the vertebral bodies in the kyphotic area bear more stress, which is consistent with the results of previous studies [34]. The anterior edge of the vertebra is a physiologically weak area [35]. The stress of the vertebral body is concentrated in front of the vertebral body in flexion. In the present study, it was found that after two-level ACDF, the peak stress on the superior and inferior vertebral bodies decreased during flexion, regardless of whether a correction was made because the plate in front of vertebral bodies provided support for the load above. Our results suggest that the maximum stress localized around the screw in C4 during lateral bending after complete correction, a finding that could imply an elevated risk of implant instability over time, potentially leading to screw loosening [36]. A previous clinical study reported an overloaded vertebral body phenomenon after multi-level ACDF [34] because the middle vertebral body with a lower elastic modulus was squeezed by the cage with a greater elastic modulus [35]. The present study demonstrated this from a biomechanical point of view. In addition, partial correction and complete correction produced greater stress on the intermediate instrumented vertebra compared with non-correction, which may cause the height of the anterior edge of the vertebral body to decrease [34].

Cervical kyphosis will cause the load of the cervical vertebra to move forward [37]; thus, the load on facet joints, as the rear load-bearing structure, will become smaller. The stress on facet joints is considered to be associated with axial pain, and a reduction in stress may lead to a decrease in neck pain for patients. In the present study, it was also observed that the facet joint stress of kyphosis segments was smaller than that of physiological lordosis. However, for adjacent segments, the facet joints of the kyphosis cervical spine bear more stress. After surgery, the peak stress of the facet joints of instrumented segments decreased, and partial correction decreased most obviously. However, our results cannot provide an explanation for the correction of the stress of adjacent facet joints.

Excessive mechanical stress will induce degeneration of the intervertebral disc [38]. Tan LA et al. [37] suggested that cervical kyphosis shifts the axial load anteriorly, thus potentially accelerating intervertebral disc degeneration. Surprisingly, it was found in the present study that IDPs of physiological lordosis cervical spine are greater than those of kyphosis in most cases. The possible reason was that the two FE models came from different individuals, and their load transfer processes may be different. The changes in IPDs of cervical kyphosis still need further study. The risk of ASD can be evaluated by the stress of the intervertebral disc [39]. Our study found that, except for C6/7 in flexion, the difference between adjacent segment IDPs before and after operation was not significant. Mumtazet al. [40] constructed cervical FE models with lordotic, straight, and kyphotic alignment and simulated intervertebral disc replacement. They found that the lordotic model showed the least IDP. The results of the present study suggested that in two-level ACDF, the IDPs of adjacent segments had little difference in different degrees of correction. Hu [16] and John [41] both constructed a kyphotic cervical FE model and reported the ROMs in flexion and extension, which were close to the results of our study.

Several limitations of the current study should be taken into account. First, we have assumed healthy material behavior of cervical spine components in our models. However, cervical kyphosis is often accompanied by degenerative and rigid changes [37]. Therefore, the model in this study may not be a perfect representative of the real-world clinical scenario. Second, because the structure of a kyphotic cervical spine may be different from normal, it is not appropriate to construct a kyphosis FE model by adjusting the curvature from physiological lordosis to kyphosis. Therefore, two individuals were selected for modeling. However, even if the influence of gender, age, and body shape was considered, there are still differences in the cervical spine in different individuals. The results with small differences between physiological lordosis and kyphosis need to be carefully reviewed. Third, the model’s simplicity, which omitted surrounding muscle tissue and utilized idealized conditions for ligaments and the annulus fibrosus, may have influenced the accuracy of our biomechanical predictions. The absence of muscle forces in our simulation could lead to an overestimation of post-surgical instability. The ligaments were represented with tension-only springs, which does not account for their complex nonlinear behavior, potentially affecting the realism of the stress distribution and stability analysis. The annulus fibrosus was modeled with a homogeneous isotropic material, which may not fully capture its anisotropic and nonlinear properties, impacting the disc’s response under various loading conditions. Fourth, surgical models of two-level ACDF in C4–6 were constructed in the present study. Further research is needed to popularize the results under different kinds of kyphosis and different surgical segments. Fifth, the present study is limited by its focus on a single case for each condition, which restricts the generalizability of the results. Given the variability in cervical spine anatomy and pathologies among different individuals, this approach may not capture the full spectrum of clinical presentations. In future studies, it is imperative to construct and analyze a variety of patient-specific models that represent a broader demographic, including different ages, genders, and levels of disease severity.

## 5. Conclusions

The FE results suggest that after two-level ACDF, the peak stress of the upper and lower instrumented vertebral bodies decreased in flexion and extension and increased in lateral bending and axial rotation regardless of correction. The stress of the middle instrumented vertebral body increased in all working conditions after surgery. Facet joint stress in kyphotic segments decreased after surgery. For complete correction, the stress of the upper instrumented vertebral body increases in lateral bending, suggesting a heightened risk of implant loosening. For correction to straighten cervical curvature, the middle instrumented vertebral body bore the greatest stress in flexion, but the stress on the facet joints of kyphotic segments was lowest compared to non-correction and complete correction. After surgery, IDPs and stress of anterior annulus fibrosus in the lower adjacent segment reduced. However, correction had little effect on the IDP of adjacent segments.

## Figures and Tables

**Table 1:** The material properties and element types of finite element models.

Material Type	Young Modulus (MPa)	Poisson’s Ratio		Element Type
Cortical bone	10,000	0.3		C3D8R
Trabecular bone	450	0.29		C3D4
Endplate	500	0.4		C3D8R
Posterior bone	3500	0.29		C3D4
Annulus fibrosus	2	0.45		C3D8R
Nucleus pulposus	1	0.49		C3D8R
Facet cartilage	10	0.4		C3D8R
Ligaments	Non-linearity		Spring Unit	
Implants	110,000	0.3		C3D4

## Data Availability

No new data were created or analyzed in this study. Data sharing is not applicable to this article.
